# Sentinel lymph node biopsy after neoadjuvant treatment of breast cancer using blue dye, radioisotope, and indocyanine green: Prospective cohort study

**DOI:** 10.1016/j.amsu.2020.09.030

**Published:** 2020-09-22

**Authors:** Prakasit Chirappapha, Tanet Chatmongkonwat, Panuwat Lertsithichai, Wiriya Pipatsakulroj, Chanika Sritara, Thongchai Sukarayothin

**Affiliations:** a Department of Surgery, Faculty of Medicine Ramathibodi Hospital, Mahidol University, Bangkok, Thailand; b Department of Pathology, Faculty of Medicine Ramathibodi Hospital, Mahidol University, Bangkok, Thailand; c Department of Radiology, Faculty of Medicine Ramathibodi Hospital, Mahidol University, Bangkok, Thailand

**Keywords:** Sentinel lymph node biopsy, Indocyanine green, Locally advanced breast cancer, Neoadjuvant chemotherapy

## Abstract

**Background:**

The breast cancer treatment paradigm has shifted to neoadjuvant treatment. There are many advantages to neoadjuvant treatment, such as tumor downsizing, *in vivo* tumor biology testing, treating micrometastasis, and achieving complete pathological response (a surrogate marker for overall survival). However, in the post neoadjuvant settings, sentinel lymph node biopsy can be done using a dual staining technique to decrease the false-negative rate (FNR) and increase the detection rate. However, many hospitals are not equipped to use radioisotopes. Here we investigate the detection rate and accuracy of sentinel lymph node biopsy in post neoadjuvant treatment breast cancer, comparing radioisotope, isosulfan blue, and indocyanine green (ICG) approaches.

**Material and methods:**

This prospective study includes breast cancer patients (T2–4, N1–2) who had received neoadjuvant treatment. Carcinomas were confirmed by tissue pathology. Patients who had previous surgical biopsy or surgery involving the axillary regions, and those with a history of allergy to ICG, isosulfan blue, or radioisotope were excluded from the study.

**Result:**

The study was done between July 1, 2019 to March 31, 2020. The mean age of participants was 53 years. Fourteen (60.87%) were post-menopause, two (8.7%) were perimenopause, and seven (30.43%) were premenopause. The clinical-stage distribution of the participants was: 2A (8.7%), 2B (34.78%), 3A (43.48%), and 3B (13.04%). The primary tumor size was 4.82 ± 2.73 cm. The lymph node size was 1.8 ± 0.96 cm. The detection rates at the individual level were 95.23% with ICG, 85.71% with isosulfan blue, and 85.71% with a radioisotope. The detection rate increased up to 100% when the ICG and blue dye methods were combined. The FNRs of sentinel lymph node biopsy at the individual level were: 10% using ICG, 30% using isosulfan blue, and 40% using radioisotope. At the lymph node level, the detection rates were 93.22% using ICG, 81.78% using isosulfan blue, and 53.87% using a radioisotope. The FNRs of sentinel lymph node biopsy at the lymph node level were 19.05% with ICG, 21.43% with isosulfan blue, and 18.03% with a radioisotope. However, the FNR was less than 10% when ICG, isosulfan blue, and a radioisotope were combined.

**Conclusion:**

We can perform sentinel lymph node biopsy by combining blue dye with ICG as an optional modality and achieve a comparable outcome with combine radioisotope in locally advanced breast cancer after neoadjuvant treatment.

## Highlights

• ICG alone has a high detection rate.93.22%
• Detection rate will increase to 96.71% when combined with blue dye.
• The accuracy of blue dye combined with ICG was statistically significant better than combine with isotope (p = 0.014).
• The combined use of blue dye with ICG is appropriate for the detection of sentinel lymph nodes in locally advanced breast cancer patients.

## 1 Introduction

There are many available modalities to perform sentinel lymph nodes (SLN) biopsies, such as using isosulfan blue, radioisotope, and indocyanine green (ICG) with a near-infrared camera. Sentinel lymph nodes can be examined to assess the axillary lymph nodes status, resulting in less morbidity than axillary lymph node dissection [[1], [2], [3]]. Past studies have found that the detection rate of sentinel lymph node biopsy using ICG was higher than when using isosulfan blue [4,5]. Other studies [6,7] have reported 94–100% detection rates of sentinel lymph nodes using ICG. The average number of lymph nodes is 2.8–3.1. Studies that used these two techniques together achieve a detection rate of 99.5% [8].

However, the above literature addresses early cancer patients and does not include locally advanced cancer patients who received neoadjuvant treatment. The study of locally advanced breast cancer patients who received neoadjuvant treatment found that the lymph node detection rate decreased from 97% to 77.6% compared to those who did not receive neoadjuvant treatment, but the accuracies of the examinations did not differ. Other studies [9] that performed sentinel lymph node biopsy in locally advanced breast cancer found that the sensitivity, false-negative rate (FNR), negative predictive value (NPV), and accuracy of sentinel lymphnode biopsy (SLNB) were 78.0%, 22.0%, 75.8%, and 87.0%, respectively.

The pattern of lymphatic drainage in patients before and after receiving neoadjuvant treatment was different, but there was at least one lymphatic tract that remained the same. This consistency confirmed that sentinel lymph node biopsy can still be performed after neoadjuvant treatment [10].

In locally advanced breast cancer patients who received neoadjuvant chemotherapy, the sentinel lymphnode biopsy detection rate before neoadjuvant treatment was 99.2%, while the detection rate reduced to 65.6% in the after neoadjuvant chemotherapy group. However, the sentinel lymph nodes will be more accurate if the two techniques were combined, using radioisotope and isosulfan blue injection. The pre-treatment rate increased from 98.8% to 99.5%, and the lymph node detection rate after neoadjuvant chemotherapy increased from 52.9% to 76.2%. The FNR reduced from 16% to 8% [11].

**Fig. 1:**
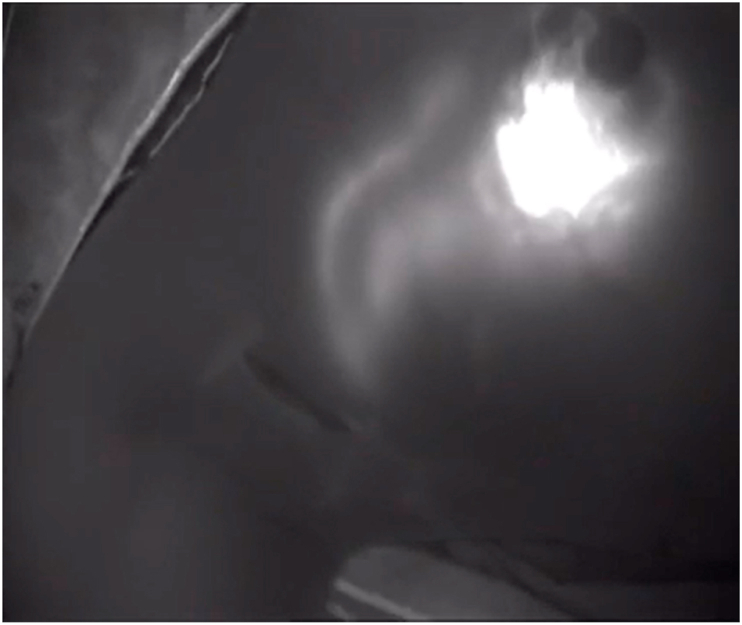
Fluorescence signal of a subcutaneous lymphatic vessel was identified by using near infrared camera. Signal disappeared beyond the lateral edge of pectoralis major muscle.

Therefore, sentinel lymph node biopsy, done before and after chemotherapy, using the combined technique will significantly increase the detection rate. However, not every hospital can perform radioisotope protocols. Radioisotope methods are relatively high cost and time-consuming and require adequate facilities for the handling and disposal of isotopes. This research is the first study to address the detection rate and accuracy of sentinel lymph node biopsy in locally advanced breast cancer patients who received neoadjuvant treatment comparing radioisotope to isosulfan blue and ICG. (see Fig. 1)

## 2 Methods

### 2.1 Study design and participant

This prospective study analyzed the detection rates and the accuracy of sentinel lymph node biopsy in breast cancer patients treated with neoadjuvant treatment from July 1, 2019 to March 31, 2020 using multiple techniques: radioisotope, isosulfan blue, and ICG with a near-infrared camera.

Informed consent was obtained from all patients and the study was approved by Institutional Review Board. The protocol had been registered at Thai Clinical Trials Registry (TCTR) with the identification number TCTR20200730003 and this research has been reported in line with the STROCSS criteria [12].

Eligible patients were those with locally advanced breast cancer (T2–4, N1–2) who received neoadjuvant treatment, with tissue pathology confirmed carcinoma. Patients who had surgical biopsy or surgery that involved the axillary regions, and those with a history of allergy to ICG, isosulfan blue, or radioisotope were excluded from the study.

**Fig. 2:**
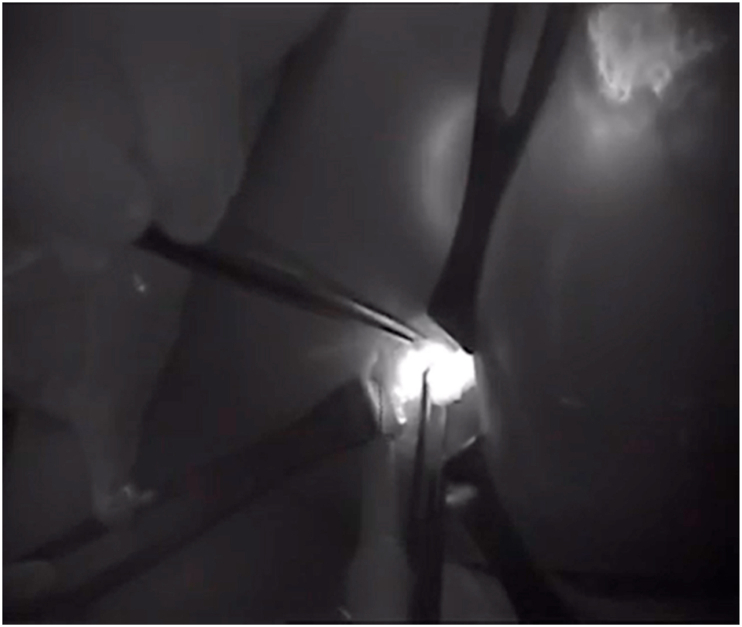
After skin incision, the sentinel lymphnode showed intense fluorescence signal.

**Fig. 3:**
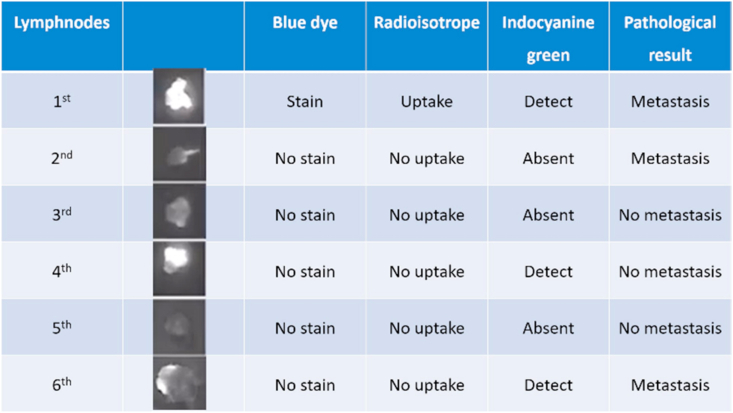
The picture show record form of harvested SLN in three modalities.

The lymph nodes were considered SLNs when we observed either a blue lymph node that uptake the blue dye, fluorescent lymph node by NIR imaging (see Fig. 2), and radioactive lymph node based on the conventional method, including the hottest node and nodes that showed more than 10% of the maximum value as counted by the gamma probe. After the detection of the SLNs, all patients proceeded to undergo further axillary lymph node dissection. The result of frozen section pathology was recorded in record form (Fig. 3).

### 2.2 Statistical methods

Statistical analysis of patients’ characteristic data was done using descriptive statistics. The identification and FNRs in locally advanced breast cancer patients treated with neoadjuvant therapy were calculated using sensitivity and specificity, and by accuracy comparing between isosulfan blue, radioisotope, and ICG findings. The detection rate was calculated by the number of successful mappings divided by the total number of mapping cases performed for each modality. All statistical analyses were performed using STATA software (version 14).

## 3 Results

### 3.1 Patient characteristics

Between July 1, 2019 to March 31, 2020, 21 patients entered the trial. The patient and tumor characteristics are shown in Table 1. The mean age was 53 years old. Fourteen (60.87%) were post-menopause, two (8.7%) were perimenopause, and seven (30.43%) were premenopause. The distribution of clinical stages were: 2A (8.7%), 2B (34.78%), 3A (43.48%), and 3B (13.04%). The mean primary tumor size was 4.82 ± 2.73 cm. The mean lymph node size was 1.8 ± 0.96 cm.

**Table 1:** Patients and tumor characteristics.

Data	Total (n = 23)
Age (years) mean ± SD	53.08 ± 10.36
Menopausal status, n(%)	
Premenopausal	7(30.43)
Perimenopausal	2(8.70)
Postmenopausal	14(60.87)
Side, n(%)	
Right breast	12(52.17)
Left breast	11(47.83)
Clinical Staging, n(%)	
2A	2(8.70)
2B	8(34.78)
3A	10(43.48)
3B	3(13.04)
Primary size (cm), mean ± SD	4.82 ± 2.73
Lymph node size, mean ± SD	1.80 ± 0.96
Histopathology of Breast, n(%)	
Invasive carcinoma non-other specified	2(8.7)
Invasive ductal carcinoma	17(73.91)
Invasive mammary carcinoma	3(13.04)
Invasive lobular carcinoma	1(4.35)
Cytology of fine needle aspiration lymph nodes, n(%)	
No metastasis	12(52.17)
Metastasis	11(47.83)

The detection rates at the individual level were 95.23% with ICG, 85.71% with isosulfan blue, and 85.71% with a radioisotope. When combining ICG and the blue dye method, the detection rate was 100% (Table 2). The FNR of sentinel lymph node biopsy at the individual level was 10% with ICG, 30% with isosulfan blue, and 40% with a radioisotope (Table 3).

**Table 2:** Sentinel lymph node detection rate according to each modality technique at the individual level and lymph nodes level.

Sentinel lymph node identification technique	Detection rate (%) Individual-level	Detection rate (%) Lymph nodes level
ICG	95.23%	93.22%
Blue dye	85.71%	81.78%
Radioisotrope	85.71%	53.87%
ICG + Blue dye	100%	96.71%
Blue dye + Radioisotrope	95.23%	84.65%
ICG + Radioisotrope	95.23%	93.84%
ICG + Blue dye + Radioisotrope	100%	97.03%

ICG, indocyanine green.

**Table 3:** The false-negative rate in each modality in sentinel lymph node biopsy at the individual level.

	False-negative rate (%)
ICG	10%
Blue dye	30%
Radioisotope	40%

ICG, indocyanine green.

At the lymph node level, the detection rate was 93.22% with ICG, 81.78% with blue dye method, and 53.87% with a radioisotope. When combined, the detection rate for ICG plus blue dye was 96.71%, 84.65% for blue dye plus radioisotope, and 93.84% ICG plus radioisotope. When using all three methods combined, the detection rate increased to 97.03% (Table 2).

The FNRs of sentinel lymph node biopsy at the lymph node level were: 19.05% with ICG, 21.43% with the isosulfan blue, and 18.03% with a radioisotope. However, the FNR was less than 10% when ICG, isosulfan blue, and the radioisotope were combined.

When the detection rate of each modality was compared to the blue dye and the radioisotope – the current standard procedure – we found that the detection of the combined technique using ICG and blue dye was significantly different from the blue dye and radioisotope (p = 0.041) (Table 4).

**Table 4:** Comparing the detection rates of each modality to the current standard procedure.

		Blue Dye + Radioisotrope		p-value	Detection rate (%)
		Positive	Negative		
ICG	Positive	13	6	0.317	13/16 (81.3%)
	Negative	3	1		
	**Total**	16	7		
Blue Dye	Positive	14	0	0.157	16/16 (87.5%)
	Negative	2	7		
	**Total**	16	7		
Radioisotope	Positive	12	0	0.045	16/16 (75.0%)
	Negative	4	7		
	**Total**	16	7		
ICG + Blue Dye	Positive	16	6	0.014	22/16 (100%)
	Negative	0	1		
	**Total**	16	7		
ICG + Radioisotrope	Positive	13	6	0.317	22/16 (81.3%)
	Negative	3	1		
	**Total**	16	7		
ICG + Blue dye + Radioisotrope	Positive	16	6	0.014	22/16 (100%)
	Negative	0	1		
	**Total**	16	7		

ICG, indocyanine green.

After the sentinel lymph node was sent to the intraoperative frozen section, we proceed to performed axillary lymph node dissection in all patients. The mean number of lymph nodes in the axillary content was 12.52 ± 5.89. In 23 patients, there were nine (39.13%) positive lymph nodes in the axillary content. The positive non-sentinel lymph node rate was 39.13%.

### 3.2 Comparison of the adverse effects

Regarding the safety for each SLN mapping tracer, no patient showed any adverse reaction or complication related to the preoperative injection for SLN mapping. All patients tolerated the procedure well without allergic reactions.

## 4 Discussion

This is the first prospective cohort study to evaluate the efficacy of sentinel lymph node detection using the ICG fluorescence method in locally advanced breast cancer patients who had received neoadjuvant treatment. The detection rate using this method was significantly higher than that of using the isosulfan blue or radioisotope method in patients with locally advanced breast cancer. This detection rate was 93.22% using ICG in a single modality, and the detection rate in the dual technique (combining ICG with isosulfan blue) was 96.71%. In the SENTINA trial, the identification rate was 80.1%, and the FNR was 14.2%, and when combining radioisotope with isosulfan blue, the FNR was just 12%. If there are more than three sentinel lymph nodes, the FNR will be 7.3%.

In the ACOZOG Z1071 trial [13], the identification rate was 92.7%, and the FNR was 12.6%, but the FNR was just 8.6% when using the dual technique. When there were more than three sentinel lymph nodes, the FNR was 9.1%.

Our data with locally advanced breast cancer patients after the neoadjuvant treatment is consistent with the literature. The identification rate when using ICG combined with isosulfan blue was 96.71%, and the FNR was 10%. The detection rate of the blue dye plus ICG was better than the blue dye plus isotope (p = 0.041). Based on our findings, we propose that the technique of combining ICG with isosulfan blue method can be applied to hospitals unable to perform sentinel lymph node biopsy using radioisotope. Moreover, the ICG technique was safer and more cost-effective than the radioisotope method, and does not require facilities for the handling and disposal of isotopes. However there are some situations that would not advocate to use this technique such as: patients who allergy to indocyanine green, matted axillary lymphnode after neoadjuvant treatment.

This study has some limitations. First, the sample size was too small to evaluate the power of each treatment modality. Second, we could not perform an axillary lymph node biopsy for all patients before neoadjuvant chemotherapy, and evaluated only clinical lymph node metastasis using sonography. The scope of this study was limited to evaluating the SLN identification rate and FNR for breast cancer patients after neoadjuvant treatment.

## 5 Conclusion

We found that ICG used alone has a high detection rate (93.22%), which is further increased (to 96.71%) when combined with blue dye. The accuracy of blue dye combined with ICG was statistically significant better than combine with isotope (p = 0.014). The combined use of blue dye with ICG is appropriate for the detection of sentinel lymph nodes in locally advanced breast cancer patients and performs as well as radioisotope approaches.

## Sources of funding

No grants or financial support were received by any of the authors in relation to this study or to the writing of this article.

## Ethical approval

Committee on Human Rights Related to Research Involving Human Subjects.

Faculty of Medicine Ramathibodi Hospital, Mahidol University.

Protocol Number: COA MURA2019/620.

## Consent

“Written informed consent was obtained from the patient for publication of this study and accompanying images. A copy of the written consent is available for review by the Editor-in-Chief of this journal on request”

## Registration of research studies

The protocol had been registered at Thai Clinical Trials Registry (TCTR). The identification number is TCTR20200730003.

## Provenance and peer review

Not commissioned, externally peer reviewed.

## CRediT authorship contribution statement

**Prakasit Chirappapha:** Writing - original draft, Data curation, Writing manuscript and interpretation of data. **Tanet Chatmongkonwat:** Formal analysis, Data curation, Correspondent, interpretation of data, and analysis. **Panuwat Lertsithichai:** Funding acquisition, Acquisition of data. **Wiriya Pipatsakulroj:** Funding acquisition, Acquisition of data. **Chanika Sritara:** Funding acquisition, Acquisition of data. **Thongchai Sukarayothin:** Acquisition of data, Funding acquisition.

## Declaration of competing interest

All authors have no any financial and personal relationships with other people or organization that could inappropriately influence (bias) their work.

## References

[1] Hennessy B.T., Hortobagyi G.N., Rouzier R., Kuerer H., Sneige N., Buzdar A.U. (2005). Outcome after pathologic complete eradication of cytologically proven breast cancer axillary node metastases following primary chemotherapy. J. Clin. Oncol..

[2] Dominici L.S., Negron Gonzalez V.M., Buzdar A.U., Lucci A., Mittendorf E.A., Le-Petross H.T. (2010). Cytologically proven axillary lymph node metastases are eradicated in patients receiving preoperative chemotherapy with concurrent trastuzumab for HER2-positive breast cancer. Cancer.

[3] Kang Y.J., Han W., Park S., You J.Y., Yi H.W., Park S. (2017). Outcome following sentinel lymph node biopsy-guided decisions in breast cancer patients with conversion from positive to negative axillary lymph nodes after neoadjuvant chemotherapy. Breast Canc. Res. Treat..

[4] Sugie T., Sawada T., Tagaya N., Kinoshita T., Yamagami K., Suwa H. (2013). Comparison of the indocyanine green fluorescence and blue dye methods in detection of sentinel lymph nodes in early-stage breast cancer. Ann. Surg Oncol..

[5] Kitai T., Kawashima M. (2012). Transcutaneous detection and direct approach to the sentinel node using axillary compression technique in ICG fluorescence-navigated sentinel node biopsy for breast cancer. Breast Canc..

[6] Abe H., Mori T., Umeda T., Tanaka M., Kawai Y., Shimizu T. (2011). Indocyanine green fluorescence imaging system for sentinel lymph node biopsies in early breast cancer patients. Surg. Today.

[7] Guo J., Yang H., Wang S., Cao Y., Liu M., Xie F. (2017). Comparison of sentinel lymph node biopsy guided by indocyanine green, blue dye, and their combination in breast cancer patients: a prospective cohort study. World J. Surg. Oncol..

[8] Lee S., Kim E.Y., Kang S.H., Kim S.W., Kim S.K., Kang K.W. (2007). Sentinel node identification rate, but not accuracy, is significantly decreased after pre-operative chemotherapy in axillary node-positive breast cancer patients. Breast Canc. Res. Treat..

[9] Park S., Park J.M., Cho J.H., Park H.S., Kim S.I., Park B.W. (2013). Sentinel lymph node biopsy after neoadjuvant chemotherapy in patients with cytologically proven node-positive breast cancer at diagnosis. Ann. Surg Oncol..

[10] Tsuyuki S., Yamaguchi A., Kawata Y., Kawaguchi K. (2015). Assessing the effects of neoadjuvant chemotherapy on lymphatic pathways to sentinel lymph nodes in cases of breast cancer: usefulness of the indocyanine green-fluorescence method. Breast.

[11] Kuehn T., Bauerfeind I., Fehm T., Fleige B., Hausschild M., Helms G. (2013). Sentinel-lymph-node biopsy in patients with breast cancer before and after neoadjuvant chemotherapy (SENTINA): a prospective, multicentre cohort study. Lancet Oncol..

[12] Agha R.A., Borrelli M.R., Vella-Baldacchino M., Thavayogan R., Orgill D.P., for the STROCSS Group (2017). The STROCSS statement: strengthening the reporting of cohort studies in surgery. Int. J. Surg..

[13] Boughey J.C., Suman V.J., Mittendorf E.A., Ahrendt G.M., Wilke L.G., Taback B. (2013). Sentinel lymph node surgery after neoadjuvant chemotherapy in patients with node-positive breast cancer: the ACOSOG Z1071 (Alliance) clinical trial. Jama.

